# The value of pancreatic stone protein in predicting acute appendicitis in patients presenting at the emergency department with abdominal pain

**DOI:** 10.1186/1471-230X-12-154

**Published:** 2012-10-25

**Authors:** Christoph Tschuor, Dimitri Aristotle Raptis, Përparim Limani, Thomas Bächler, Christian Eugen Oberkofler, Stefan Breitenstein, Rolf Graf

**Affiliations:** 1 Department of Surgery, University Hospital Zurich, Raemistrasse 100, Zurich, CH-8091, Switzerland

**Keywords:** PSP, Pancreatic stone protein, Acute appendicitis, Abdominal pain

## Abstract

**Background:**

Pancreatic Stone Protein (PSP) is a protein naturally produced mainly in the pancreas and the gut. There is evidence from experimental and clinical trials that blood PSP levels rise in the presence of inflammation or infection. However, it is not known whether PSP is superior to other established blood tests (e.g. White Blood Count, Neutrophils or C - reactive protein) in predicting appendicitis in patients presenting with abdominal pain and a clinical suspicion of appendicitis at the emergency room.

**Methods/design:**

The PSP Appendix Trial is a prospective, multi-center, cohort study to assess the value of PSP in the diagnostic workup of acute appendicitis. 245 patients will be prospectively recruited. Interim analysis will be performed once 123 patients are recruited. The primary endpoint of the study concerns the diagnostic accuracy of PSP in predicting acute appendicitis and therefore the evidence of appendicitis on the histopathological specimen after appendectomy.

**Discussion:**

The PSP Appendix Trial is a prospective, multi-center, cohort study to assess the value of PSP in the diagnostic workup of acute appendicitis.

**Trial registration:**

ClinicalTrials.gov: NCT01610193; Institution Ethical Board Approval ID: KEKZH- Nr. 2011–0501

## Background

Appendicitis is defined as an inflammation of the vermiform appendix and is a common condition and an urgent surgical illness. It is one of the most common causes of acute abdominal pain and remains a diagnostic challenge. It has variable manifestations and generous overlap with other clinical syndromes. In fact morbidity increases significantly with the diagnostic delay and left untreated, appendicitis has the potential for severe complications, including perforation or sepsis, and may even cause death. Despite diagnostic and therapeutic advancement in medicine, appendicitis still remains a clinical emergency [1].

No single sign, symptom, or diagnostic test exists which accurately confirms an appendiceal inflammation. The classic history of anorexia and periumbilical pain followed by nausea, right lower quadrant (RLQ) pain, and vomiting occurs in only 50% of cases [1,2].

Accepted etiologic factors of appendicitis are appendiceal lumen obstruction and infection. The main therapeutic option still remains the surgical removal of the vermiform appendix even though a few studies promote an antibiotic treatment [3]. After admission to the emergency department (ED), patients presenting with abdominal pain caused by a variety of different etiologic factors have to be worked up with the goal of approaching 100% sensitivity for the diagnosis in a time-, cost-, and consultation-efficient manner [4].

It is reported that appendectomy carries a complication rate of up to 15%, as well as increased associated costs and the discomfort of hospitalisation and surgery [5]. Delay in diagnosis and treatment account for much of the associated mortality and morbidity. Therefore, the goal of the surgeon is to diagnose appendicitis as early as possible.

The overall mortality rate of 0.2 - 0.8% is attributable to complications of the disease rather than to surgical interventions [2]. The mortality rate in children ranges from 0.1% to 1%; in patients older than 70 years, the rate rises above 20%, primarily because of diagnostic and therapeutic delay [4,6].

Appendiceal perforation is associated with increased morbidity and mortality compared with non-perforating appendicitis. The mortality risk of acute but not gangrenous appendicitis is less than 0.1%, but the risk rises to 0.6% in gangrenous appendicitis. The rate of perforation varies from 16% to 40%, with a higher frequency occurring in patients below 50 years (40-57%) and in patients older than 50 years (55-70%), in whom misdiagnosis and delayed diagnosis are common. Complications occur in 1-5% of patients with appendicitis. Postoperative wound infections account for almost one third of the associated morbidity [1,6,7].

PSP is a secretory protein produced predominantly in the pancreas and the gut. There is evidence from experimental and clinical trials that the levels of PSP in the blood increase in the presence of inflammation or infection [8,9]. However, it is still unknown whether PSP is superior to other established blood tests (e.g. WBC or CRP) in predicting appendicitis in patients presenting with abdominal pain and a clinical suspicion of appendicitis at the emergency room.

The objective of this trial is to 1) determine the diagnostic accuracy of PSP in predicting acute appendicitis and 2) compare PSP with other established markers used in the diagnostic work-up.

## Methods/design

The PSP Appendix Trial is a prospective, multi-center, cohort study to assess the value of PSP in the diagnostic workup of acute appendicitis. Two hundred and forty five patients will be recruited. Interim analysis will be performed once 123 patients are recruited [ Additional file 1]. A power analysis will be performed on the actual and precise data collected. At interim analysis the potential need to modify the sample size will be investigated. If any changes are suggested by the external data monitoring committee, the principal investigators will decide on the feasibility of the potential changes and submit a formal addendum to the ethics committee. No changes will be made to the protocol or study design unless first approved by the ethics committee. Any changes to the protocol approved by the ethics committee will be updated at clinicaltrials.gov [NCT01610193].

### Study objectives

The primary endpoint of the study is histopathological diagnosis of appendicitis on the specimen after appendectomy in order to assess the diagnostic accuracy of PSP calculated in predicting acute appendicitis. Secondary endpoints concern:

1. Grade of appendicitis detected intraoperatively:

G0: Appendix without any abnormalities detected introperatively

G1: Acute appendicitis

G2: Gangrenous appendicitis

G3: Perforated or phlegmon

G4: Periappendicular abscess

2. Alvarado Score elements which are obtained from the patient’s history, the physical examination and from laboratory tests. A score below 5 is strongly against a diagnosis of appendicitis, while a score of 7 or more is strongly predictive of acute appendicitis. In patients with an equivocal score of 5 or 6, a CT scan is used to further reduce the rate of negative appendectomy [10].

3. Pancreatic Stone Protein (PSP) blood serum/plasma levels will be correlated with this score. Surgical complications defined according to the Clavien-Dindo Classification will be monitored [11].

### Inclusion criteria

All patients that will present at the emergency department with abdominal pain and a clinical suspicion of acute appendicitis:

Age >18 years of age

Clinical suspicion of appendicitis as the primary or differential diagnoses

Patients able to provide informed consent

### Exclusion criteria

Age <18 years of

Abdominal discomfort without tenderness or rebound or clinical suspicion of appendicitis

Pregnancy

Patients with impaired consciousness

Patients not able to provide informed consent (Non-German Speakers)

Patients that will receive an appendectomy as part of another elective procedure

Patients who are unable to understand the study purpose.

Family members of study investigators or employees of the participating centers

### Participating surgeons and clinics

Doctors in the Emergency Room who have the first contact with the patients are interns, junior and senior residents as well as chief residents and attending surgeons of the Department of Surgery. Operating surgeons are senior residents, chief residents, attending surgeons as well as consultant surgeons. Participating centers are the Division of Visceral and Transplantation Surgery of the University Hospital Zurich as well as affiliated clinics in Switzerland. Several European hospitals will be participating in this trial as well.

### Data collection and statistics

The assessment for patient recruitment starts in the very beginning as soon as the patient attends the emergency room (ER), receives a consultation and a doctor’s assessment of the patient’s condition Additional file 2. If there is a clinical suspicion of appendicitis, the patient should be assessed for eligibility (inclusion and exclusion criteria) to participate in the PSP Appendicitis Trial Additional file 3. If any patient is assessed for eligibility of inclusion in this trial but was found to meet any of the exclusion criteria, the reason and explanation of the exclusion should be also documented in the Clinical Trial Management System (CTMS) https://www.PSPtrail.com[12].

Once a patient is assessed for eligibility and is found to meet the inclusion criteria, the patient should immediately enter the trial before any investigations have ordered blood tests, imaging, etc. The consent forms and patient information have to be provided to the patient. If the patient does not agree to participate in the trial, this should be documented in the online CTMS. If the patient agrees to participate in this trial, blood has to be taken for Pancreatic Stone Protein (PSP) measurement (1 tube of 5-10 ml, for serum determination in clinical chemistry). This tube has to be labelled as B1 followed by the patient trial number (B1_II_YYYY). The letter “B” stands for blood” and “1” as the first blood collection. The patient trial number should be the name initials (II) followed by the year of birth (YYYY). As part of the standard care, blood has to be taken for analysis for full blood count and CRP measurement at participating institutions. In particular, the White Blood Cell Count (WBC), Neutrophil Granulocytes (%) and C-reactive Protein (CRP) will have to be entered into the CTMS. The blood has to be taken at the same time for PSP, WBC and CRP as soon as the patient signs the consent form while still at the emergency department. A pregnancy test (beta hCG) has to be also performed and information entered into the CTMS. The investigators are free to perform any other blood tests deemed necessary.

The investigators are free to use any kind of imaging as part of the routine management of patients. Ultrasound Scan (USS) or Computed Tomography (CT) data will be entered in the CTMS.

The physician will then decide whether the patient should be subject to further diagnostics, referred to another inpatient or outpatient department or be discharged home [ Additional file 4]. If the patient is admitted for an operation, this has also to be documented in the CTMS. If the physician decides to take blood to assess the progress of the patient before an operation or discharge, an additional tube for PSP measurement is taken (see above). This tube has to be labelled as (B2_II_YYYY, e.g. B2_DR1978, see example above, “2” stands for second blood test for PSP). The same centrifugation process has to be performed to sample B2 as for B1.

Then the investigators will be asked to indicate the type of the operation and the intra-operative findings, if the patient was operated. During hospitalisation, the physician should document any complication according to the Clavien-Dindo Classification of surgical complications. For the patients that were operated, the physician will need to document the date of discharge. Once the histopathology report of the resected specimen has been received, the diagnosis will be entered in the CTMS. To ensure standardized reporting, all centers will adhere to the above classification of appendicitis.

Continuous variables will be compared with the Student t, Mann–Whitney U, one-way ANOVA, and Kruskal-Wallis tests, where appropriate. Differences among proportions derived from categorical data will be compared using the Fischer’s Exact or the Pearson χ^2^ tests, where appropriate. All p values will be two-sided and considered statistically significant if p ≤ 0.05. Sensitivity, specificity, accuracy, positive predictive value (PPV), negative predictive value (NPV), positive likelihood ratio (PLR), negative likelihood ratio (NLR), Yuden’s index (YI), diagnostic odds ratio (OR), and the receiver operator characteristic (ROC) curve will be calculated. Data will be presented as mean (SD), median (i.q.r.) and Odds Ratios (95% CI) where appropriate. Reproducibility of PSP measurements will be performed by duplicating the samples and the variability assessed by the Pearson’s Correlation Coefficient. Statistical analysis will be performed using SPSS Statistics version 20 (SPSS: An IBM Company, Chicago IL, 2011).

### Ethics

This study is conducted in accordance with the principles of the Declaration of Helsinki and ‘good clinical practice’ guidelines. The independent medical ethics committee of canton Zurich (Kantonale Ethikkommission Zürich, Switzerland) has approved the study protocol.

## Discussion

The surgeon’s aim is to correctly diagnose the appendicitis and therefore to minimize the negative appendectomy rate without increasing the incidence of perforation [13]. He has to evaluate a diverse group of patients who present at the ED with abdominal pain and has to consider different aetiologies [14]. The goal of a diagnostic accuracy approaching 100% sensitivity and specificity should be reached in a time-, cost-, and consultation-efficient manner [4].

The overall mortality rate of 0.2-0.8% is attributable to complications of the disease rather than to surgical interventions [2]. The mortality rate in children ranges from 0.1% to 1%; in patients older than 70 years, the rate rises above 20%, primarily because of diagnostic and therapeutic delay [4,6]. Since delayed diagnosis and treatment accounts for much of the mortality and morbidity associated with appendicitis, it is the goal of the surgeon to make an accurate diagnosis as early as possible. False positive diagnosis leads to unnecessary operations which are estimated at 5-15%, while false negative diagnosis may lead to a delay that increases the chances for perforation, peritonitis and sepsis.

Keeping in mind, that appendectomy carries a complication rate of up to 15%, as well as associated costs and the discomfort of hospitalization and surgery, a reliable diagnostic tool such as blood test would be of utmost advantage [5].

## Conclusion

The PSP Appendix Trial is a prospective, multi-center, cohort study to assess the value of PSP in the diagnostic workup of acute appendicitis.

## Abbreviations

PSP: Pancreatic stone protein; RLQ: Right lower quadrant; ED: Emergency department; WBC: White blood count; CRP: C-reactive protein; CTMS: Clinical trial management system; CT: Computed tomography; USS: Ultrasound scan.

## Competing interests

The authors report the following conflict of interest: Drs. Raptis, Tschuor, Limani, Oberkofler and Breitenstein have reported that no potential conflicts of interest exist with any companies/organizations whose products or services may be discussed in this article. Prof. Graf is inventor and the University of Zurich owns the patent for PSP/reg as a marker of sepsis.

## Authors' contributions

CT and DAR drafted the manuscript. CEO, PL and RG designed the protocol and co-authored the writing of the manuscript. DAR performed the study design and calculation of the sample size of the study. All other authors participated in the design of the study and are local investigators. All authors were involved in editing the manuscript. All authors read and approved the final manuscript.

## Study approval

This study is approved by the Medical Ethics Commission of the Canton Zurich, Switzerland via a peer reviewed process (Kantonale Ethikkommission Zurich). ClinicalTrials.gov: NCT01610193; Institution Ethics Board Approval ID: KEKZH- Nr. 2011–0501.

## Pre-publication history

The pre-publication history for this paper can be accessed here:

http://www.biomedcentral.com/1471-230X/12/154/prepub

## Supplementary Material

Additional file 1 Sample Chart. Click here for file

Additional file 2 Patient Journey Flow Chart. Click here for file

Additional file 3 Case Report Form – New Case – ER. Click here for file

Additional file 4 Case Report Form – Admission. Click here for file
