# Supplementary material for: The value of pancreatic stone protein in predicting acute appendicitis in patients presenting at the emergency department with abdominal pain
Source: BMC Gastroenterol. 2012 Oct 25;12:154. doi: 10.1186/1471-230X-12-154 (PMC3503734; doi:10.1186/1471-230X-12-154)
Supplement: Additional file 1 — Sample Chart. [file 1471-230X-12-154-S1.pdf]

Additional file 1: Sample Size Calculation Flow Chart

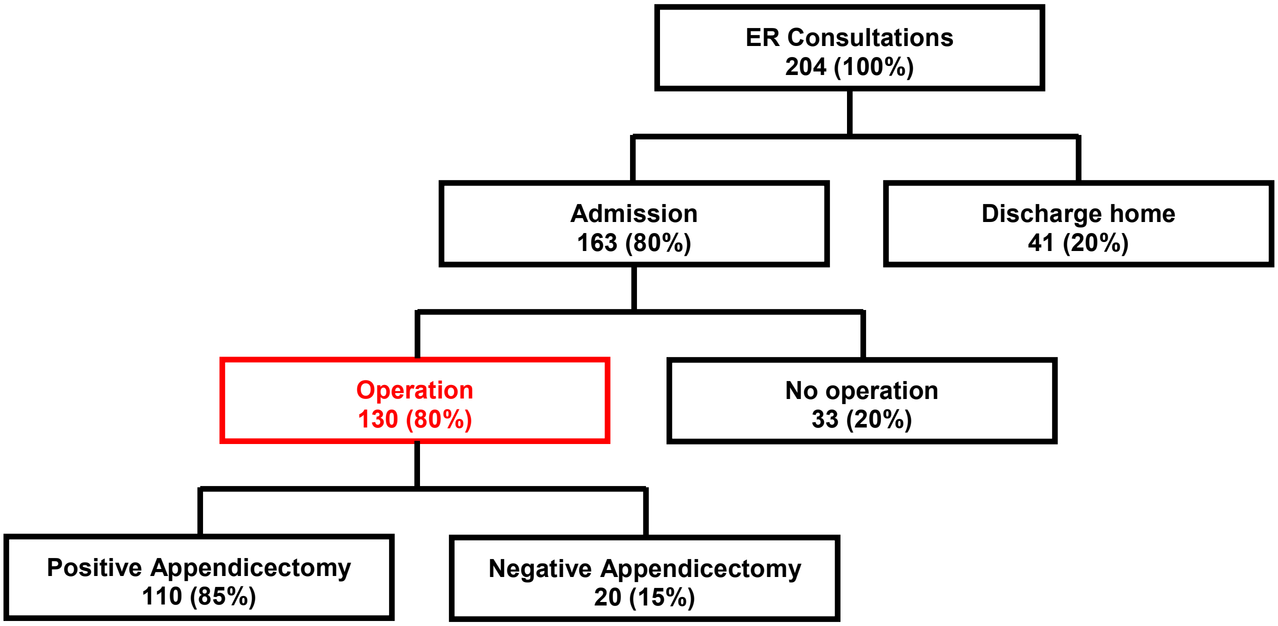

**Test:**	t tests - Means: Difference between two independent means (two groups)	
**Analysis:**	A priori: Compute required sample size	
**Input:**	**Tail(s)**	= Two
	**Effect size d**	= 0.7905694
	**α err prob**	= 0.05
	**Power (1-β err prob)**	= 0.90
	**Allocation ratio N2/N1**	= 0.185
**Output:**	**Noncentrality parameter δ**	= 3.2522181
	**Critical t**	= 1.9786708
	**Df**	= 128
	**Sample size group 1**	= 110
	**Sample size group 2**	= 20
	**Total sample size**	= 130
	**Actual power**	= 0.8975579

Oversampling – 20%: lost to follow up patients, missing data/blood samples

Total number of patients attending A&E: 245

Interim analysis will be performed once 123 patients are recruited. A power analysis will be performed and the sample size re-calculated based on the actual and precise data collected. At interim analysis, the external data monitoring committee will decide upon the continuation or discontinuation of the trial, as well the potential need to modify the sample size. If any changes are suggested by the external data monitoring committee, the principal investigators will decide on the feasibility of the potential changes and submit a formal addendum to the ethics committee. No changes will be made to the protocol or study design unless first approved by the ethics committee.
