# Supplementary figures and images for: The value of pancreatic stone protein in predicting acute appendicitis in patients presenting at the emergency department with abdominal pain
Source: BMC Gastroenterol. 2012 Oct 25;12:154. doi: 10.1186/1471-230X-12-154 (PMC3503734; doi:10.1186/1471-230X-12-154)

## Additional file 2: Patient Journey Flow Chart

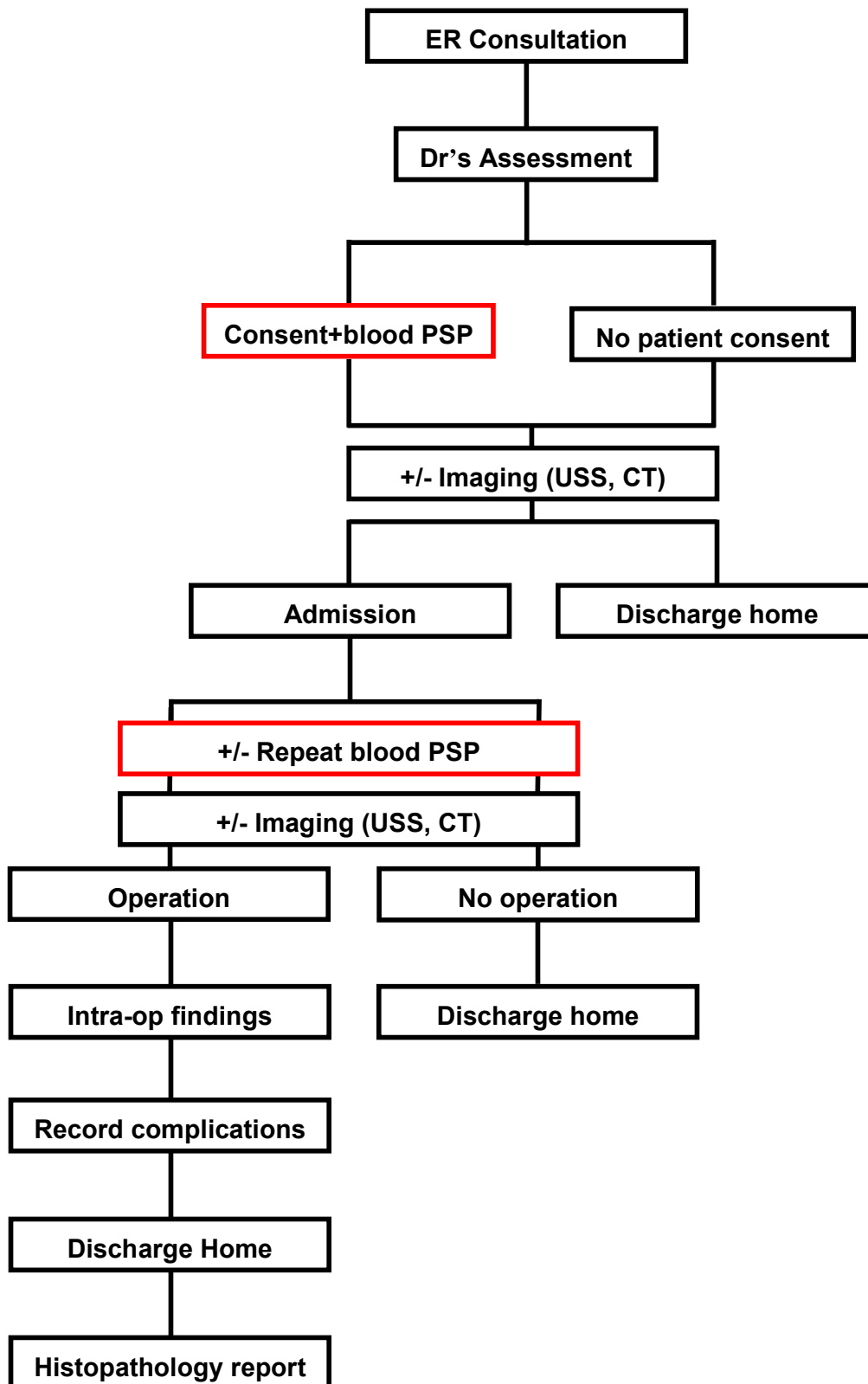

Supplement: Additional file 2 — Patient Journey Flow Chart. [file 1471-230X-12-154-S2.pdf]
