# Supplementary material for: The value of pancreatic stone protein in predicting acute appendicitis in patients presenting at the emergency department with abdominal pain
Source: BMC Gastroenterol. 2012 Oct 25;12:154. doi: 10.1186/1471-230X-12-154 (PMC3503734; doi:10.1186/1471-230X-12-154)
Supplement: Additional file 3 — Case Report Form – New Case – ER. [file 1471-230X-12-154-S3.pdf]

Additional file 3: Case Report Form – New Case – ER

**ER Consultation**	Date and time _ _ /_ _/ _ _ _ _, _ _:_ _ (dd/mm/yyyy, hh:mm)	
**Doctor's Last Name**	____	
**Demographics**	Date of birth _ _ /_ _/ _ _ _ _ (dd/mm/yyyy)	
Patient Hospital Number:	____	
Gender	Male ☐, Female ☐	
Ethnicity	Caucasian	☐
	Asian	☐
	African	☐
	Other/mix _____________________________________	☐
Comorbidities	Cardiovascular	☐
	Respiratory	☐
	Neurological	☐
	Diabetes Mellitus	☐
	Rheumatological	☐
	Other:____	☐
**Suspicion of appendicitis**	Yes	☐
	No	☐
**Alvarado score**	Abdominal pain that migrates to the right iliac fossa	☐
	Anorexia (loss of appetite) or ketones in the urine	☐
	Nausea or vomiting	☐
	Tenderness (Pain on pressure) in right iliac fossa	☐
	Rebound tenderness	☐
	Raised temperature (≥37.3℃)	☐
	Leukocytosis (>10 WCC x10^9/L)	☐
	Neutrophils >75% (left shift)	☐
	Time from symptoms to ER consultation	____ hours
**Consent obtained**	Yes	☐
	No	☐
**Blood obtained for PSP**	Yes	☐
	No	☐
	Date and time: _ _ /_ _/ _ _ _ _, _ _:_ _ (dd/mm/yyyy, hh:mm)	
**Re-Attendance to ER**	Yes	☐
	No	☐
**Patient status**	Admitted for appendectomy/observation/referred	☐
	Discharged home directly from the Emergency Room	☐
