# Supplementary material for: The value of pancreatic stone protein in predicting acute appendicitis in patients presenting at the emergency department with abdominal pain
Source: BMC Gastroenterol. 2012 Oct 25;12:154. doi: 10.1186/1471-230X-12-154 (PMC3503734; doi:10.1186/1471-230X-12-154)
Supplement: Additional file 4 — Case Report Form – Admission. [file 1471-230X-12-154-S4.pdf]

## Addtional file 4: Case Report Form – Admission

| | | |
|---|---|---|
| **ER Attendance bloods** | White blood cell count (WBC): _________(x10$^9$/L) | |
| | Neutrophil granulocytes (% of WCC): ________% | |
| | CRP: ________________(mg/l) | |
| **Ultrasound Scan** | Yes | ☐ |
| | No | ☐ |
| | Date and time: _ _ /_ _/ _ _ _ _, _ _:_ _ (dd/mm/yyyy, hh:mm) | |
| | No abnormalities ☐, Visualized ☐, Thickened ☐, free fluid ☐ | |
| | Other:____________________________________________ | ☐ |
| **CT** | Yes | ☐ |
| | No | ☐ |
| | Date and time: _ _ /_ _/ _ _ _ _, _ _:_ _ (dd/mm/yyyy, hh:mm) | |
| | No abnormalities ☐, Visualized ☐, fluid ☐, fat stranding ☐, dilatation ☐, wall thickened ☐, fecalith ☐, perforation ☐ | |
| | Other:____________________________________________ | ☐ |
| **Pre-op repeat blood - PSP** | Yes | ☐ |
| | No | ☐ |
| | Date and time: _ _ /_ _/ _ _ _ _, _ _:_ _ (dd/mm/yyyy, hh:mm) | |
| **Operation** | Yes | ☐ |
| | No | ☐ |
| | Date and time: _ _ /_ _/ _ _ _ _, _ _:_ _ (dd/mm/yyyy, hh:mm) | |
| | Open (McBurney's incision ☐), (Midline laparotomy ☐) | ☐ |
| | Laparoscopic | ☐ |
| | Laparoscopic converted to open (McBurney's incision) | ☐ |
| | Laparoscopic converted to open (Midline laparotomy) | ☐ |
| | Other:_______________________________________________ | ☐ |
| **Intra-op findings** | Appendix normal | ☐ |
| | Acute appendicitis | ☐ |
| | Gangrenous appendicitis | ☐ |
| | Perforated appendicitis or phlegmon | ☐ |
| | Periappendicular abscess | ☐ |
| | Other:______________________________________________ | ☐ |
| **Complications** | No complications | ☐ |
| | Grade I | ☐ |
| | Grade II (medications only) | ☐ |
| | Grade IIIa (intervention under local anesthetic) | ☐ |
| | Grade IIIb (intervention under general anesthesia) | ☐ |
| | Grade IVa (organ failure, admission to ICU) | ☐ |
| | Grade IVb (multi-organ failure, admission to ICU) | ☐ |
| | Grade V (Death) | ☐ |
| **Type of complication** | Highest grade:___________________________________ | |
| **Date of discharge** | Yes, with operation ☐, without operation ☐ | ☐ |
| | Date and time: _ _ /_ _/ _ _ _ _, _ _:_ _ (dd/mm/yyyy, hh:mm) | |
| | No (death): _ _ /_ _/ _ _ _ _, _ _:_ _ (dd/mm/yyyy, hh:mm) | ☐ |
| **Histopathology report** | Appendix normal | ☐ |
| | Acute appendicitis | ☐ |
| | Gangrenous appendicitis | ☐ |
| | Perforated appendicitis or phlegmon | ☐ |
| | Periappendicular abscess | ☐ |
| | Other:_______________________________________________ | ☐ |
